# Isolation, Identification, and Evaluation of the Pathogenicity of a Porcine Enterovirus G Isolated From China

**DOI:** 10.3389/fvets.2021.712679

**Published:** 2021-07-22

**Authors:** Xue Mi, Chunjie Yang, Ying Lu, Hejie Wang, Qiuying Qin, Ronglin Chen, Zhenkong Chen, Yunyan Luo, Ying Chen, Zuzhang Wei, Weijian Huang, Kang Ouyang

**Affiliations:** College of Animal Science and Technology, Guangxi University, Nanning, China

**Keywords:** Enterovirus G, virus isolation, phylogenetic analysis, pathogenicity, piglets

## Abstract

Enterovirus G (EV-G) infects porcine populations worldwide and the infections are generally asymptomatic, with the insertion of the papain-like cysteine protease gene (PLCP) increasing the potential public health threats. However, the genetic and pathogenic characteristics of EV-G itself are not fully understood as yet. In the present study, one EV-G strain, named CH/17GXQZ/2017, was isolated and purified from piglets with diarrheic symptoms from the Guangxi Province, China. This strain produced stable cytopathic effects on Marc-145 cells with a titer of 5 × 10^6^ PFU/mL. The spherical enterovirus particles with diameters of 25–30 nm were observed by using transmission electron microscopy. The whole genome sequence of the CH/17GXQZ/2017 strain consists of 7,364 nucleotides, and the phylogenetic tree based on the amino acid sequences of VP1 indicated this strain was clustered to the G1 genotype. Seven-day-old piglets were inoculated orally with the CH/17GXQZ/2017 strain in order to evaluate its pathogenicity. Although none of the infected piglets died during the experiment, clinical neurological symptoms were observed manifesting as mild hyperemia and Nissl bodies vacuolization in the cerebrum. In addition, the infection with the CH/17GXQZ/2017 strain decelerated the weight gain of suckling piglets significantly. This study demonstrates that CH/17GXQZ/2017 is pathogenic to neonatal piglets and advance knowledge on the biological characteristics, evolution and pathogenicity of EV-G.

## Introduction

Enterovirus species include viruses that infect humans (species A~D), bovine (species E and F), swine (species G) and non-human primates (A, B, D, H, and J) (1, 2). Porcine enteroviruses (PEV) were originally divided into 13 serotypes (PEV-1 to−13) (3, 4), and then subdivided into three genera *Teschovirus, Sapelovirus* and *Enterovirus* (5, 6). PEV-1 to 7 and PEV-11 to 13 were reclassified to the genus *Teschovirus*, PEV-8 to the genus *Sapelovirus* (5, 7), and PEV-9 and PEV-10 were reclassified as *Enterovirus G* (EV-G) (8, 9). EV-G is the member of the family *Picornaviridae* of the order *Picornavirales*, which currently comprises 20 genotypes (EV-G-1 to−20) (10– 14) (https://www.picornaviridae.com/sg3/enterovirus/ev-g/ev-g.htm).

EV-G is a non-enveloped virus with a small positive-sense single stranded RNA genome. The genome of EV-G is ~7,400–7,500 nucleotides (nt) and possesses a single open reading frame (ORF) flanked by untranslated regions. The ORF is processed proteolytically into four structural proteins (VP1, VP2, VP3, and VP4) and seven non-structural proteins (2Apro, 2B, 2C, 3A, 3B, 3Cpro, and 3Dpol) (14–17). The UTR at both ends of the genome are required for enteroviral RNA initiation. The 5′-UTR is ~700–825 nt in length, containing secondary structural elements which are important for RNA replication as well as an internal ribosomal entry site (IRES) for the initiation of translation (18). The 3′-UTR consists of 75 to 100 nt and contains complex cis-acting elements essential for RNA replication (19).

EV-G infects porcine populations worldwide and the infection is generally asymptomatic (20). Although piglets inoculated orally with EV-G positive fecal samples can cause flaccid paralysis of the hind limbs (21), there is little evidence to confirm the correlation between EV-G infection and clinical diarrhea (11). Novel EV-G1 variants carrying the porcine torovirus (ToV) papain-like cysteine protease gene (PLCP) in the 2C/3A junction region of their genomes were first found in fecal samples from pigs with diarrheic diseases in the USA (22). There is increasing evidence to suggest that the lateral acquisition of the PLCP gene might represent a novel host immune control strategy for EV-Gs to establish a pathogenic potential under certain circumstances, and the recombination between EV-G and ToV implies there is a risk of cross-species transmission (23).

The prototype of EV-G was isolated in 1973 and 1975 from the UK (24). It is relatively small and spherical with a diameter of ~25–30 nm. It occurs without cysts and the nucleocapsid has an icosahedral symmetry (25). Most of the complete genome sequences of EV-G were obtained by next generation sequencing (NGS) (22, 26, 27), whereas studies regarding virus isolation is limited. Recently, the insertion of PLCP were shown to cause changes in the innate immune signal of EV-G, but the evolutionary genetic and pathogenic characteristics of EV-G itself are not yet fully understood. In the present study, one EV-G strain, named CH/17GXQZ/2017, was isolated and purified. The genetic characteristics as well as the pathogenesis of the CH/17GXQZ/2017 strain in 7-day-old piglets was investigated.

## Materials and Methods

### Specimen Collection and Cell Cultures

In November 2017, stool specimens were obtained from piglets with diarrhea were collected from a farm in Qinzhou City, Guangxi Province, China. Marc-145 cells were grown in Minimum Essential Medium (MEM) supplemented with 10% fetal bovine serum (FBS) (Biological Industries, Kibbutz Beit Haemek, Israel) and antibiotics (100 units/mL of penicillin and 100 units /mL of streptomycin) at 37°C in a humidified incubator in an atmosphere of 5% CO_2_.

### Detection of Samples

The stool samples were diluted 5 times in phosphate buffered saline (PBS) and the supernatants were collected after being vortexed and centrifuged at 12,000 × g at 4°C for 10 min. Total RNA was extracted from the supernatants by using a Viral DNA/RNA Miniprep Kit (Axygen Scientific, Union City, USA). RT-PCR was used to detect porcine epidemic diarrhea virus (PEDV), transmissible gastroenteritis virus (TGEV), porcine rotavirus (PoRV), porcine delta coronavirus (PDCoV), porcine kobuvirus (PKV) and EV-G, in all the samples collected and only EV-G was found. The samples were then passed through 0.22 μm filters (Millipore, Billerica, MA, USA) and the filtrates were used for virus isolation.

### Virus Isolation

EV-G was propagated in Marc-145 cells and the virus particles were isolated using trypsin as previously described (13), with minor modifications. Briefly, the monolayers of Marc-145 cells were grown in six-well plates. Growth media were then removed and washed three times with PBS before inoculation. Five hundred microliter of sample inoculum was adsorbed for 60 min at 37°C, EV-G growth medium consisting of MEM supplemented with antibiotics (100 units/mL of penicillin and 100 μg/mL of streptomycin) and 0.5 μg/mL of trypsin was added without removing the inoculum. Cells were incubated at 37°C in a 5% CO_2_ atmosphere, and examined daily for cytopathic effects (CPE). When CPE appeared in more than 80% of cells (~5 days after inoculation), the cells were harvested and subjected to three freeze-thaw cycles and then stored at −80°C.

### Plaque Assay

The EV-G isolates were purified by using a plaque method in Marc-145 cells. The cell monolayers were infected with 200 μL of a 10-fold dilution of virus particles for 1 h at 37°C, and shaken gently every 15 min. Then the virus inoculum was removed and washed three times with PBS, following which it was overlaid with 2 mL of MEM containing 1% agarose and 0.5 μg/mL trypsin. The overlaid medium was solidified by keep the cell plates at room temperature (~20°C) for 20 min. The plates were then inverted and incubated at 37°C in a 5% CO_2_ incubator. After 4 days of incubation, the cells were fixed in 10% formaldehyde for 12 h, and the cells were stained with crystal violet for the plaques to be visualized. The strain of EV-G, subsequently named CH/17GXQZ/2017, was successfully obtained after three series of purification by the plaque assay.

### Immune Serum Preparation and Indirect Immunofluorescence Assay

Since most picornaviruses are transmitted *via* the oral route (28), and it was demonstrated that antibody levels in serum were higher in mice immunized with live virus orally compared to peritoneally (29), the EV-G immune serum was prepared by oral administration of the live virus. Three-month-old Kunming mice were orally inoculated with 5 × 10^5^ PFU of EV-G strain CH/17GXQZ/2017 in a volume of 100 μL as previously described (29, 30) with a few modifications. Serum was collected at 21 days post-infection (dpi) and stored at −80°C. Non-immune sera were obtained from naïve mice. This was subsequently used for the IFA and immunohistochemistry (IHC) test.

Marc-145 cell monolayers were inoculated with EV-G CH/17GXQZ/2017 strain at a multiplicity of infection (MOI) of 0.01. At 48 h post-inoculation (hpi), cells were washed three times with PBS, followed by fixation in cold acetone at −4°C for 30 min. The cells were washed three times with PBS and then blocked with 1% BSA (Roche, Mannheim, Germany) for 60 min at room temperature (~20°C). After being washed with PBS, the cells were incubated with the mouse anti-EV-G serum as prepared above (1:100) for 1 h at 37°C. Then the cells were washed with PBS three times followed by incubation with Alexa Fluor® 488 conjugated goat anti-mouse IgG (H + L) (Abcam Inc., USA) (1:4,000) for 1 h at 37°C. The cells were subsequently washed five times with PBS. Finally, images were captured using an inverted fluorescence microscope (Nikon, Tokyo, Japan).

### Viral Replication Kinetics in Marc-145 Cells

Marc-145 cell monolayers grown in 12-well plates were infected with EV-G CH/17GXQZ/2017 strain at 0.01 MOI. After adsorption at 37°C for 60 min, MEM containing 0.5 μg/mL trypsin was added, and the plates were incubated at 37°C with 5% CO_2_. Cell supernatants were harvested in triplicates at 6, 12, 24, 36, 48, 60, 72, and 84 hpi, by centrifugation at 12,000 × g for 10 min at 4°C, and used for virus titration by using the plaque assay. Virus titration at each time points was carried out in three replicates.

### Transmission Electron Microscope Observation of the Virus

Marc-145 cells were infected with EV-G CH/17GXQZ/2017 strain at 0.01 MOI, and when the CPE was more than 80%, the cell culture supernatants were collected by centrifugation at 10,000 rpm at 4°C for 1 h. The supernatants were filtered through 0.22 μm filters and mixed with 10% polyethylene glycol 8,000 (Solarbio, Beijing, China), precipitated and stirred gently at 4°C overnight. The virus particles were precipitated by centrifugation at 12,000 rpm at 4°C for 2 h, and re-suspended with 1 mL of Tris-buffered saline solution (Solarbio, Beijing, China). Then negative staining with 2% phosphotungstic acid was performed and these were imaged using a transmission electron microscope (Hitachi TEM system, Japan, HT7800).

### Sequencing and Data Analysis

The whole genome of EV-G stain CH/17GXQZ/2017 was obtained from three overlapping fragments which were amplified by using three pairs of primers. The primers have been described previously with some of modifications (10) (Table 1). The viral genomic RNA was extracted from the EV-G stock samples using the Axy Prep™ Viral DNA/RNA Miniprep Kit (OMEGA, USA) according to the manufacturer's instructions (31). The cDNA was synthesized in a total volume of 25 μL containing 16 μL of RNA solution, 2 μL of dNTP mixture, 0.5 μL of reverse transcriptase M-MLV, 0.5 μL of RNasin inhibitor, 1 μL of oligo (dT) or the reverse primer, and 5 μL of 5 × reverse transcriptase M-MLV buffer. The mixture was incubated at 42°C for 60 min and then chilled on ice to destroy the RNA secondary structure. PCR was performed using the PrimeSTAR Max DNA Polymerase (TaKaRa, Dalian, China). The size of PCR products was checked by electrophoresis in 1.0% agarose gel after staining with GelRedTM (Biotum Inc., USA) and visualization by using an UVItec imager (Bio-Rad Laboratories Inc., USA). The expected DNA band was purified and cloned into pEASY-Blunt Cloning vector (TransGen, Beijing, China) and sequenced by Beijing Genomics Institute, Shenzhen.

**Table 1:** The primers used for complete genome amplification of EV-G.

**Primer**	**Nucleotide sequences (5^**′**^ → 3^**′**^)**	**Position**
EV-G1F	TTAAAACAGCCTGTGGGTTGTTCCCAC	1–27
EV-G1R	ACTGAACTCTGGAGCCCAAAGTCC	2,219–2,242
EV-G2F	ATGTTTACTGGGTCATTCAT	2,110–2,129
EV-G2R	CATCAAAATGGTCACTATCTG	4,541–4,561
EV-G3F	GGATACATACAGTTCAAG	4,393–4,410
EV-G3R	ACACCCCATCCGGTGGGT	7,369–7,387

The chromatograms were checked and edited by the SeqMan program. The nucleotide sequences were aligned by the MegAlign software (Lasergene, DNASTAR Inc., USA). A phylogenetic tree was constructed by using the maximum likelihood method in MEGA5.0 software.

### Quantitative Real-Time PCR

Primers were designed (forward primer: 5′-CTTGTGCATCCGGTTATGCC-3′ and reverse primer: 5′-GCAAGCAGGCACAGAGAACGC-3′) using Primer Premier 5.0 software. The recombinant plasmid was constructed by cloning the PCR fragments into a pMD18-T vector (TaKaRa, Dalian, China) and calculating the resulting copy numbers. A 10-fold series of diluted recombinant plasmids in triplicates with known copy numbers were used as templates to generated a standard curve for quantitative PCR. The reaction mixture for qRT-PCR contained 2 μL cDNA, 10 μL TB GreenTM Premix Ex TaqTM II (Takara, Dalian, China), 1.0 μL (10 pmol/μL) of each primer and ddH2O. Reactions were performed in triplicates. The amplification conditions were as follows: 95°C for 2 min, 40 cycles of 95°C for 15 s, 60°C for 1 min and a melting-curve analysis was included in the end. The qRT-PCR was performed in 96-well plates by using a LightCycler 96 (Roche, Mannheim, Germany).

### Experimental Infection of 7-Day-Old Piglets

A total of eight 7-day-old piglets (Duroc × Landrace × Yorkshire) which were nucleotide negative for foot-and-mouth disease virus (FMDV), classical swine fever virus (CSFV), porcine reproductive and respiratory syndrome virus (PRRSV), pseudorabies virus (PRV), PEDV, TGEV, PoRV, and EV-G were purchased from a commercial farm. Piglets of either sex were randomly assigned to two groups of four piglets. The piglets in the infected group (*n* = 4) were orally inoculated with 2 mL of the EV-G CH/17GXQZ/2017 strain with titers of 5 × 10^6^ PFU/mL. The same amount of cell culture media was inoculated to the piglet in mock group and these were used as controls. Milk powder (Shenzhen Premixinve Nutrition Co. Ltd., China) were used to feed the animals four times a day and they were allowed water, *ad libitum*. Clinical observations, weight and rectal temperatures were recorded daily from 0 to 7 dpi.

Fecal swabs were collected at hpi of 0, 12, 24, 36, 48, 60, 72, 84, 96, 108, 120, 132, 144, 156, and 168. Pigs were euthanized at 7 dpi and the tissues of the brain, spinal cord, stomach, duodenum, jejunum, ileum, cecum and colon were collected at necropsy. Tissues were homogenized at a concentration of 0.1 g/mL which were used for viral load detection by qPCR. Some tissue samples were also immediately fixed in 10% neutral buffered formalin for subsequent histological examination. According to previous studies (32) with minor modification, the fixed brain tissue sections were trimmed, processed and embedded in paraffin wax, stained with Nissl and then analyzed for histopathological changes. Diluted mouse anti-EV-G serum (1:100) was used for the detection of EV-G antigen with goat anti-mouse IgG conjugated to horseradish peroxidase (HRP) (Servicebio, China) (1:200) being used as secondary antibody. The animal study was reviewed and approved by the Animal Care and Welfare Committee of Guangxi University (Permission Number: GXU2020-021).

### Statistical Analysis

All the values are expressed as the means ± standard deviations (SD) or standard error of the means (SEM) of four piglets (33). Data were analyzed using the two-tailed *t*-test. Statistically significant was set to a *P*-value of 0.05.

## Results

### Isolation and Purification of EV-G Strain

**Figure 1:**
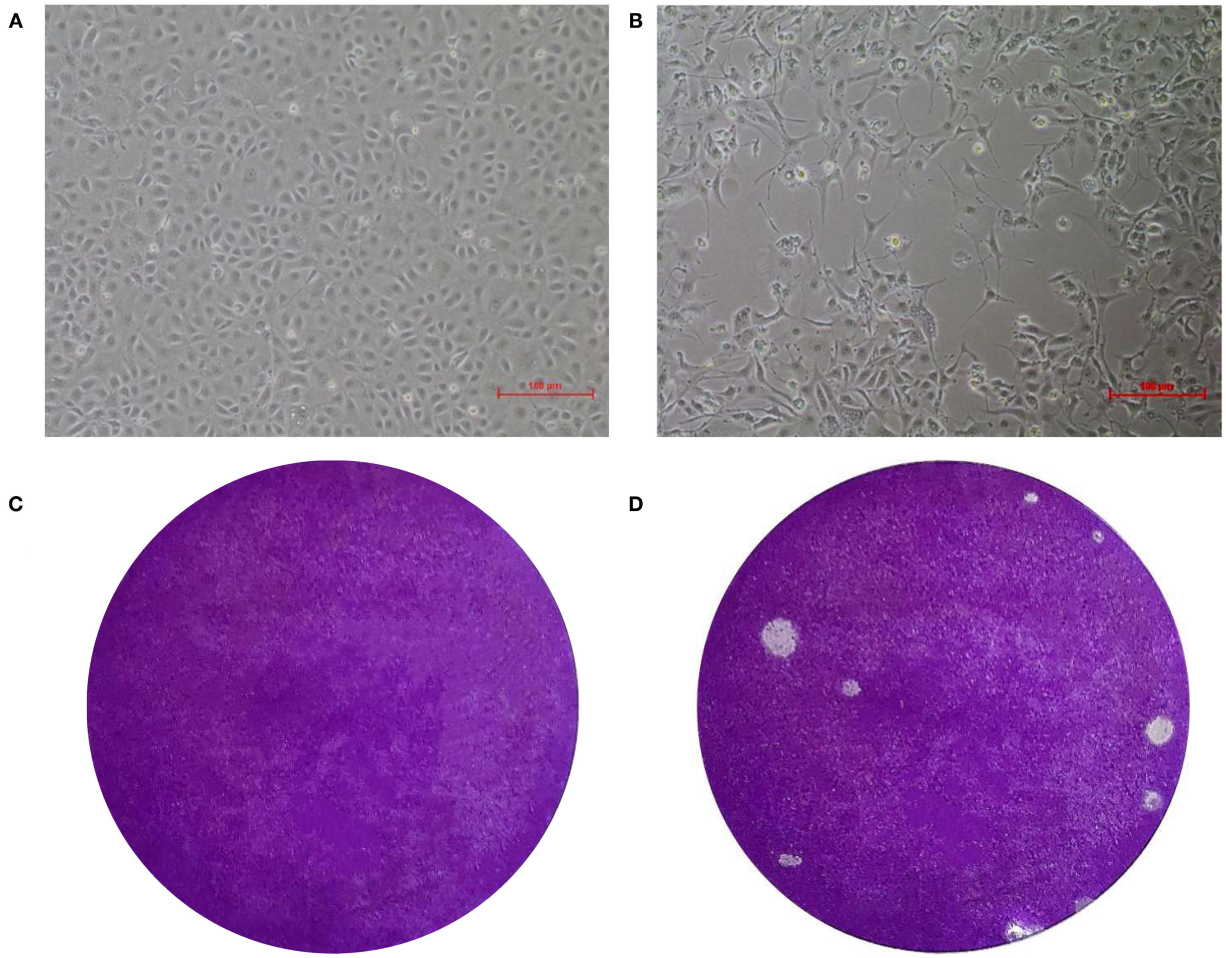
CPE and plaque formation of the EV-G CH/17GXQZ/2017 strain. Neither CPE nor viral plaques were observed in the mock cells **(A,C)**. CPE caused by the EV-G CH/17GXQZ/2017 strain in Marc-145 cells was characterized by cell shrinkage, rounding and detachment at 48 hpi (200 × magnification) **(B)**. Viral plaques of EV-G isolates of CH/17GXQZ/2017 developed at 4 dpi **(D)**.

At passage 4, typical CPE of Marc-145 cells was characterized by cell shrinking into round structures. In addition, cell layer splitting and shedding were observed at 48 hpi (Figure 1B). The virus was purified by subjecting the samples to three series of plaque assays (Figure 1D). Neither CPE nor viral plaques were observed in the mock cells (Figures 1A,C) One strain of EV-G, named CH/17GXQZ/2017, was successfully isolated, and the estimated titer was 5 × 10^6^ PFU/mL.

### Immunofluorescence Assay and Biological Characteristics of the EV-G CH/17GXQZ/2017 Strain

**Figure 2:**
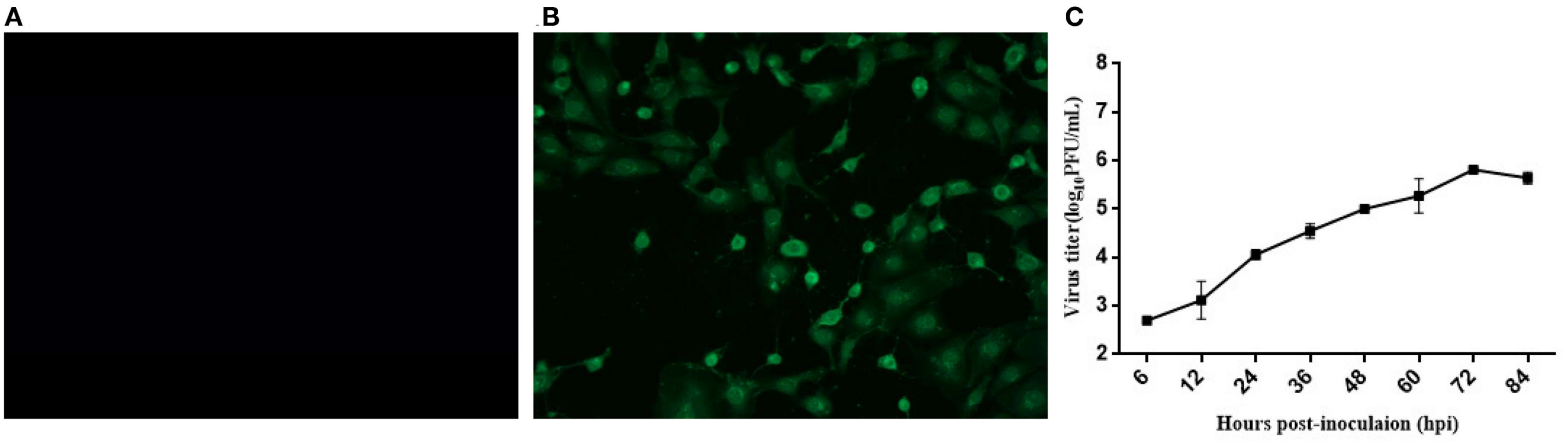
Immunofluorescence assay and growth kinetics of the EV-G CH/17GXQZ/2017 strain in Marc-145 cells. Negative control **(A)**. IFA staining in Marc-145 cells infected with the EV-G CH/17GXQZ/2017 strain at 48 hpi with mouse anti-EV-G serum (1:100) and Alexa Fluor® 488 conjugated goat anti-mouse IgG (H + L) (1:4,000) which were used as primary and secondary antibodies, respectively. This shows that the specific green fluorescence was mainly localized in the cytoplasm of infected cells (200 × magnification) **(B)**. Replication kinetics of the EV-G CH/17GXQZ/2017 strain in Marc-145 cells was determined by plaque assays. Marc-145 cells were inoculated with the EV-G CH/17GXQZ/2017 strain at 0.01 MOI, and the cell culture supernatants were harvested at 6, 12, 24, 36, 48, 60, 72, and 84 hpi, separately. The virus titers are expressed as PFU per milliliter and are shown as the mean values with standard deviations from three replicates **(C)**.

Specific green fluorescence was seen in Marc-145 cells infected with the EV-G CH/17GXQZ/2017 strain by using an IFA (Figure 2B). This indicated that the EV-G CH/17GXQZ/2017 strain could react with the specific mouse anti-EV-G serum (Figure 2A). No green fluorescence was observed using normal mouse serum control or in the mock cells.

Marc-145 cells were infected with the EV-G CH/17GXQZ/2017 strain at 0.01 MOI and the growth curve of the virus was determined (Figure 2C). The mean titers of three independent measurements at each time point at 6, 12, 24, 36, 48, 60, 72, and 84 hpi were measured. The number of viral plaques of strain CH/17GXQZ/2017 continued to rise from 6 to 72 hpi, and reached a peak at 72 hpi with a titer of 6.5 × 10^5^ PFU/mL.

**Figure 3:**
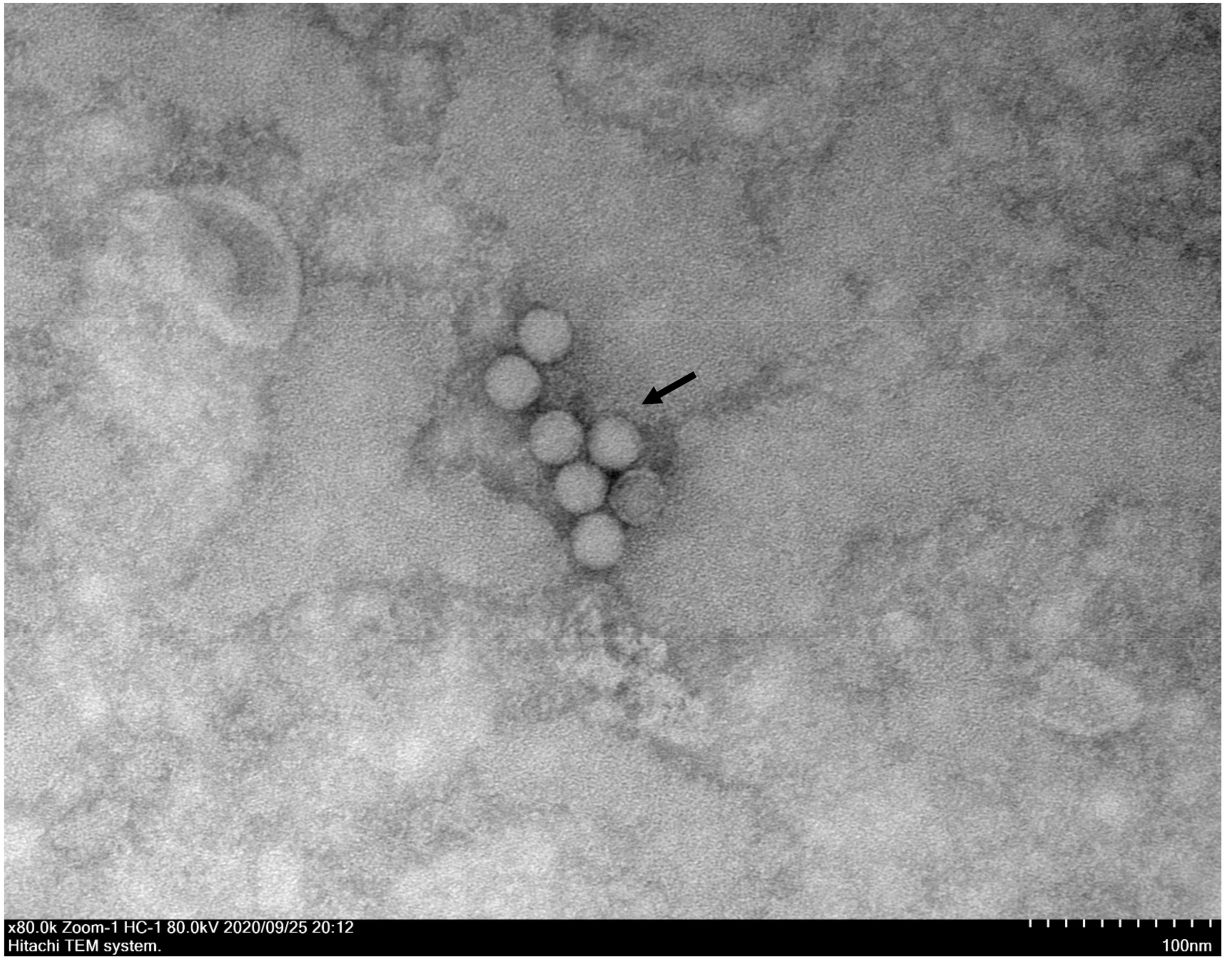
A transmission electron microscopic image of purified EV-G particles. Spherical virions with a diameter of 25–30 nm were observed and marked with a black arrow. The sample was negatively stained with 2% phosphotungstic acid. Scale bar indicates 100 nm divisions.

A typical *Picornavirales* morphology was observed for the EV-G CH/17GXQZ/2017 strain when the virus particles were observed through TEM. They appeared small and spherical and without cysts. Their average diameter was ~25–30 nm (Figure 3).

### Whole Genome Amplification of the EV-G CH/17GXQZ/2017 Strain

**Figure 4:**
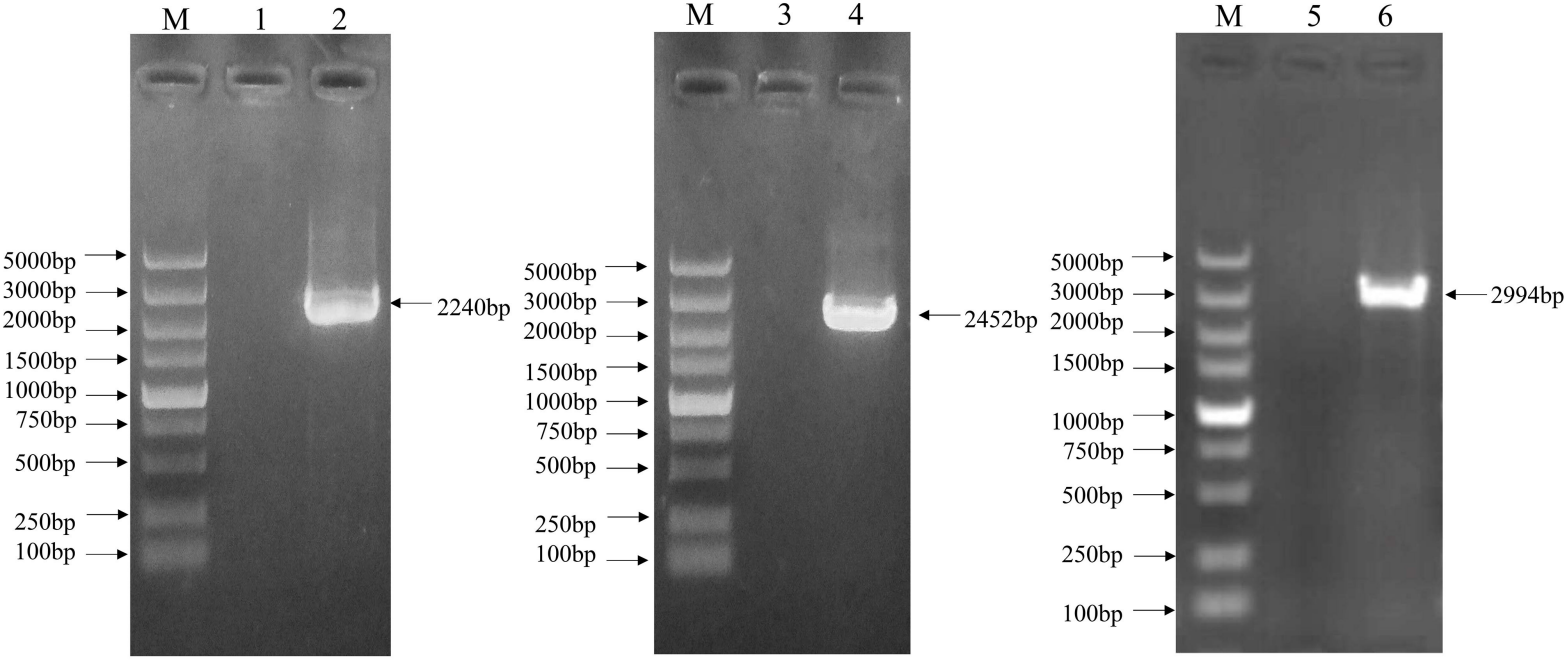
The complete genome identification of the EV-G CH/17GXQZ/2017 strain by RT-PCR. Amplification of the complete genome of the EV-G CH/17GXQZ/2017 strain. Lane M, DNA marker DL5000; Lanes 1, 3, and 5, negative controls; Lanes 2, 4, and 6, three overlapping PCR products of viral genome from the EV-G CH/17GXQZ/2017 strain.

The complete genome sequence of the EV-G CH/17GXQZ/2017 strain was successfully obtained from three overlapping fragments by RT-PCR analysis (Figure 4). The complete genome size of the CH/17GXQZ/2017 strain was 7,364-nt and consisted of 821-nt of 5′-UTR, 72-nt of 3′-UTR and 6,471-nt of ORF, the ORF maps between positions 822 and 7,292 and encoded a 2,157-amino-acid polyprotein. The CH/17GXQZ/2017 strain sequence was deposited in the GenBank database under the Accession Number, MT274669.

### Sequence Analysis of the EV-G CH/17GXQZ/2017 Strain

The nucleotide and amino acid sequence identities of the 5′-UTR, VP4, VP3, VP2, VP1, 2A, 2B, 2C, 3A, 3B, 3C, 3D, and 3′-UTR of CH/17GXQZ/2017 were compared with the strains of UKG/410/73, KOR/KUN-1811/2018/G1-PLCP and EVG/Porcine/USA/Texas2/2014/G1-PLpro (Table 2). The genome organization of CH/17GXQZ/2017 is similar to the prototype the EV-G UKG/410/73 strain, and it shows whole genome nucleotide similarities of 79.8, 77.9, and 77.2% to the strains of UKG/410/73, KOR/KUN-1811/2018/G1-PLCP and EVG/Porcine/USA/Texas2/2014/G1-PLpro, respectively.

**Table 2:** Nucleotide and amino acid identities of CH/17GXQZ/2017 when compared with EV-G reference strains.

**Gene**	**CH/17QZ/2017 (MT274669)**		**UKG/410/73 (Y14459)**				**KOR/KUN-1811/2018/G1-PLCP (MH663501)**				**EVG/Porcine/USA/Texas2/2014/G1-PLpro(KY498017)**			
	**Size** ** (nt)**	**Size** ** (aa)**	**Size** ** (nt)**	**Identity** ** (%)**	**Size** ** (aa)**	**Identity** ** (%)**	**Size** ** (nt)**	**Identity** ** (%)**	**Size** ** (aa)**	**Identity** ** (%)**	**Size** ** (nt)**	**Identity** ** (%)**	**Size** ** (aa)**	**Identity** ** (%)**
5′UTR	821	/	772	85.9	/	/	813	90.3	/	/	796	84.6	/	/
1A (VP4)	207	69	187	77.5	62	85.5	207	77.8	69	89.9	207	76.8	69	82.6
1B (VP2)	738	246	738	73.4	246	91.1	738	74.1	246	90.7	738	74.9	246	90.2
1C (VP3)	795	265	831	69.7	277	83.8	831	72.5	277	82.3	714	77.2	238	91.6
1D (VP1)	729	243	729	73.7	243	80.2	729	74.5	243	84.4	846	71.7	282	81.6
2A	450	150	450	84.9	150	81.8	450	82.2	150	92.0	450	81.1	150	93.3
2B	297	99	297	77.1	99	93.9	297	72.1	99	80.8	297	68.7	99	80.8
2C	987	329	987	82.0	329	92.4	987	77.7	329	90.3	987	78.9	329	90.3
PLCP	/	/	/	/	/	/	594	/	198	/	669	/	223	/
3A	267	89	267	77.2	89	94.4	267	72.7	89	86.5	267	74.5	89	88.8
3B	66	22	66	80.3	22	100	66	84.8	22	95.5	66	80.3	22	95.5
3C	549	183	549	82.3	183	96.2	549	72.7	183	84.2	549	71.8	183	84.2
3D	1,383	461	1,383	82.3	461	94.4	1,383	78.6	461	87.6	1,383	77.9	461	81.1
3′UTR	72	/	72	92.0	/	/	71	85.5	/	/	27	84.6	/	/
Total	7,364	/	7,351	79.8	/	/	7,985	77.9	/	/	7,999	77.2	/	/

Compared to strain UKG/410/73, the strain of CH/17GXQZ/2017 had only 80.2% amino acid homology in the VP1 gene, and it had 36 nucleotide deletions in the area of 2488~2529bp in the VP3 gene and 21 nucleotide insertions in the area of 1009~1028bp in the VP4 gene, respectively. The 3B gene of EV-G was found to be relatively conservative, and it exhibited the highest amino acid homology of up to 100% when compared with UKG/410/73, 95.5% identity when compared to the strains of KOR/KUN-1811/2018/G1-PLCP and EVG/Porcine/USA/Texas2/2014/G1-PLpro, respectively.

The strain of KOR/KUN-1811/2018/G1-PLCP and EVG/Porcine/USA/Texas2/2014/ G1-PLpro had 594 and 669 bp PLCP gene insertions at the 2C/3A junction region, respectively. Although there was no PLCP insertion in the strain of CH/17GXQZ/2017, it is noteworthy that the length of 5′-UTR of CH/17GXQZ/2017 was slightly longer than all three reference strains, due to the continuous insertion of the thirty-two A bases at position of 687–718 bp.

### Phylogenetic Analysis of the EV-G CH/17GXQZ/2017 Strain

**Figure 5:**
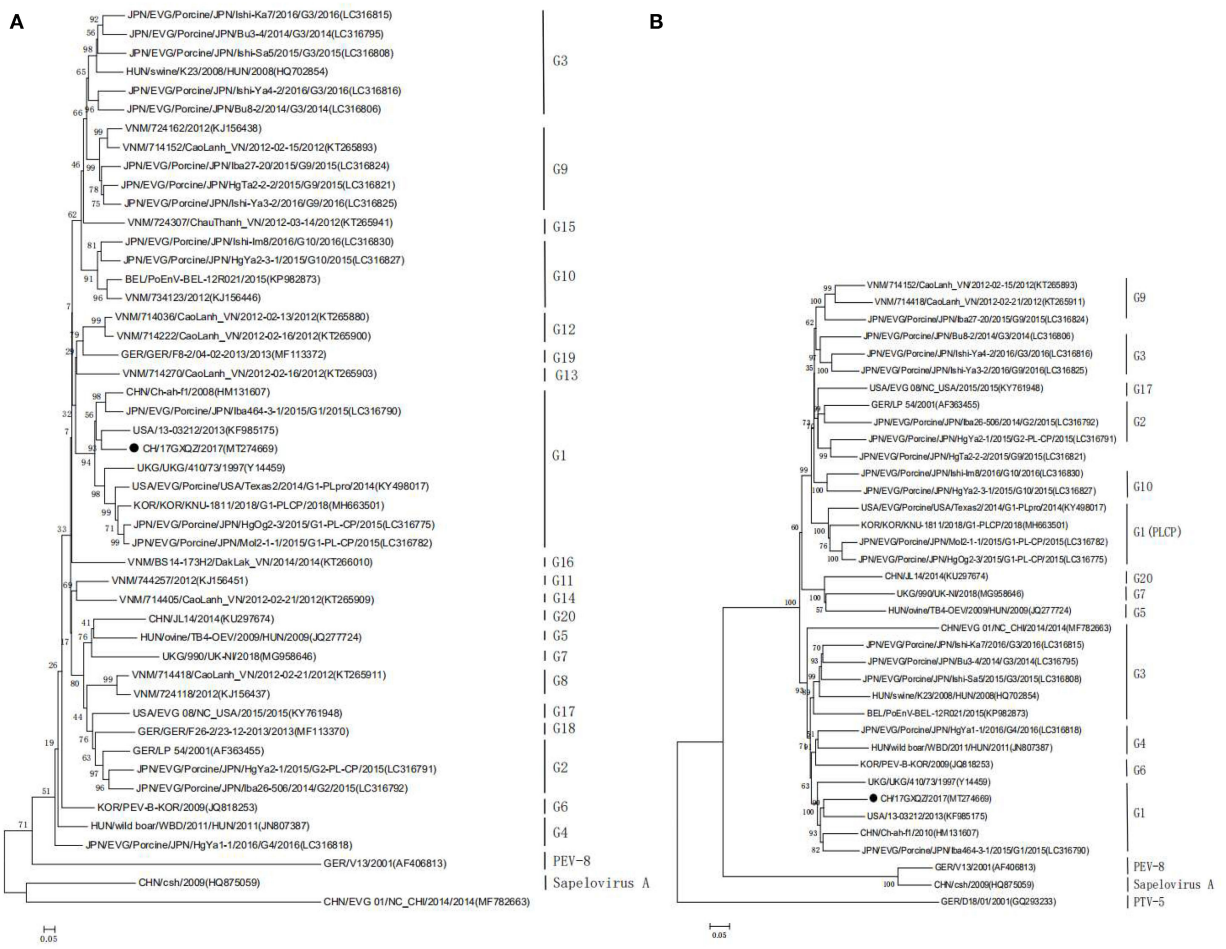
Phylogenetic analyses of the EV-G CH/17GXQZ/2017 strain. Phylogenetic analyses based on the amino acid sequences **(A)** of VP1 and the complete genome sequences **(B)** of the EV-G CH/17GXQZ/2017 strain with reference EV-G sequences obtained from the GenBank. The phylogenetic trees were constructed using the maximum likelihood method in MEGA 5.0 software. Bootstrap analysis was carried out on 1,000 replicate datasets and values are indicated adjacent to the branching points. The genetic distances were calculated using the Tamura-Nei model. The EV-G CH/17GXQZ/2017 strain obtained in this study is indicated by solid circles. The scale bars indicate the amino acid or nucleotide substitutions at each site.

EV-G classification is solely based on sequence identities of the VP1 gene and the phylogenetic tree based on the VP1 gene amino acid sequences showed that the CH/17GXQZ/2017 strain was clustered into the G1 subtype (Figure 5A). Phylogenetic analysis based on the complete genome nucleotide sequences revealed that the four strains (KY498017, MH663501, LC316782, and LC316775) were clustered to a separate group of G1 type due to the insertions of PLCP (Figure 5B). This indicated that the strain of CH/17GXQZ/2017 had a closer genetic relationship with the other three G1 type strains, USA/13-03212/2013 (KF985175), CHN/Ch-ah-f1/2010 (HM131607), and EVG/Porcine/JPN/Iba464-3-1/2015/G1 (LC316790), with the overall nucleotide similarities of 82.9, 82.8, and 81.8%, respectively.

### Pathogenicity of the EV-G CH/17GXQZ/2017 Strain on 7-Day-Old Piglets

In the virus inoculated group, all four piglets exhibited trembling motions of the hind limbs at 2 dpi, which persisted for 1–2 days. Two piglets developed neurological symptoms which manifested as excitation and they spun around in circles at 5–6 dpi. One piglet developed mild diarrhea at 4–7 dpi. No piglets exhibited any clinical signs in the mock group.

**Figure 6:**
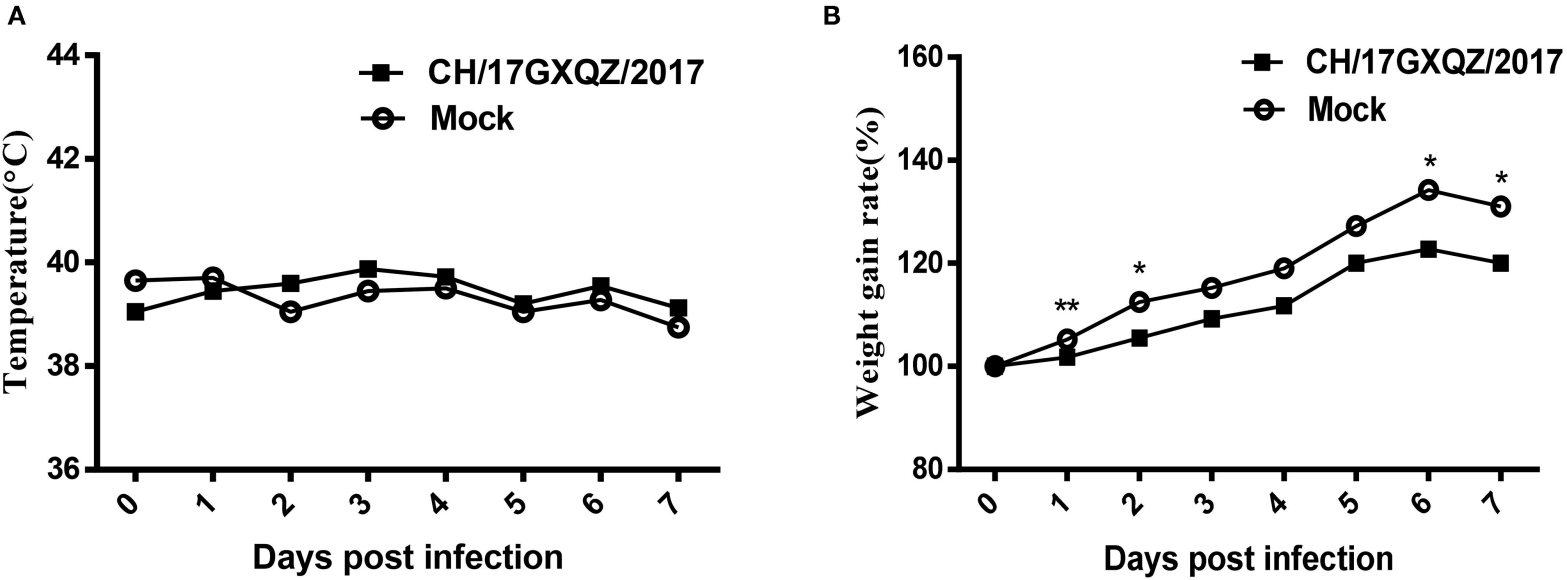
Trends of rectal temperatures and weight gain of 7-day-old piglets inoculated with EV-G. Normal rectal temperatures were present in both the infected and mock groups **(A)**. The weight gain rates of piglets were analyzed, those in the infected group was significant less than in pigs from the mock group at 1, 2, 6, and 7 dpi. Each point on the graph represents the average values from the four pigs. * and ** indicate *P* < 0.05 and *P* < 0.01, respectively, between the two groups **(B)**.

All piglets had normal rectal temperatures at 0–7 dpi (Figure 6A). Piglets in the CH/17GXQZ/2017 infected group gained less weight than those in the mock group. The weight gain rate in piglets from the infected group was significant less than those in the mock group at 1, 2, 6, and 7 dpi, with 3.5, 7, 11.5, and 11% less weight gain rate, respectively (Figure 6B).

**Figure 7:**
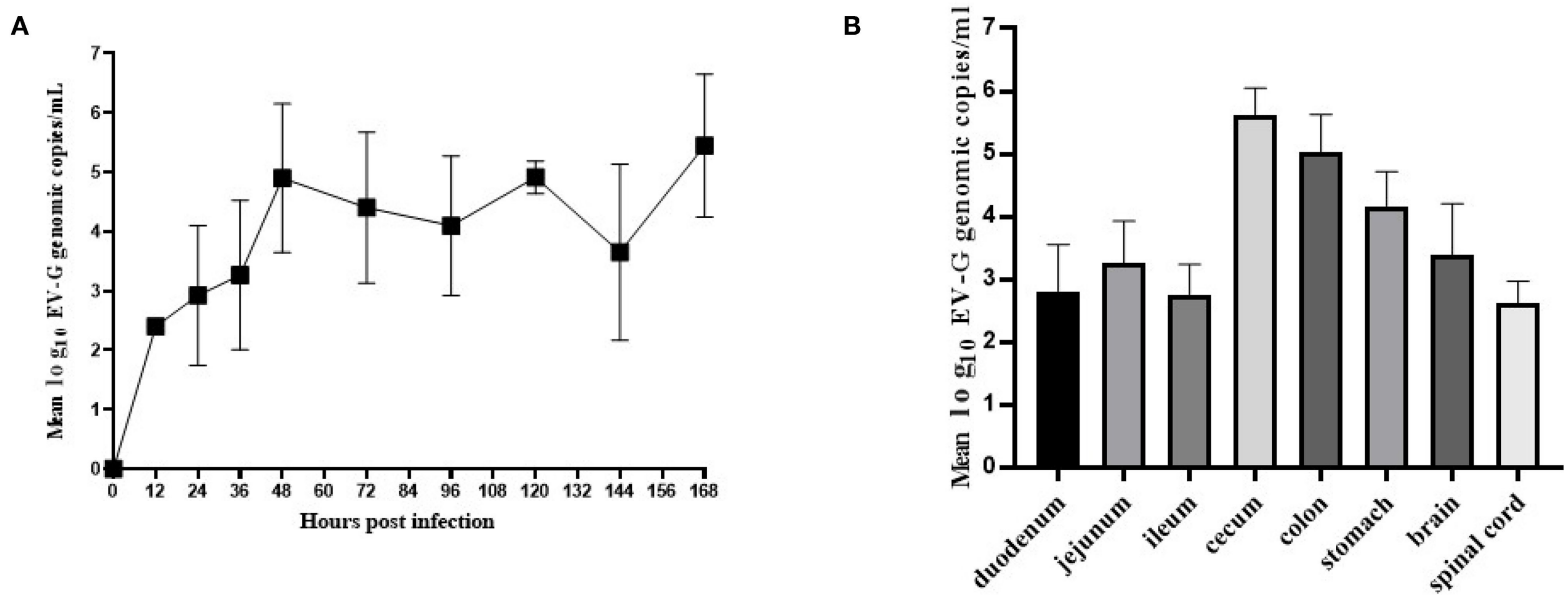
The viral shedding in fecal swabs and the viral distribution in different tissues in EV-G infected piglets. The fecal swabs were collected from piglets at 0–168 hpi. The EV-G shedding in fecal swab samples from infected piglets were detected **(A)**. Intestinal segments and other tissues (stomach, brain, spinal cord) were collected at 7 dpi. The EV-G viral RNA loads were detected **(B)**. Each point or histogram shown are the mean values ± SD (error bars) from four piglets.

Fecal swab samples were collected from which the viral copies were detected by qRT-PCR. The results showed that genomic copies of EV-G increased rapidly between 12 and 48 h and the viral load was at 10^4.9^-10^5.3^ copies/mL within 48–168 h (Figure 7A). EV-G genomic copies in the tissue samples of the brain, spinal marrow, stomach, duodenum, jejunum, ileum, cecum and colon were detected, and these showed that the genomic copies of EV-G in cecum and colon were 10^5.6^ and 10^5.1^ copies/mL, respectively, which were magnitudes higher than those in the small intestine (Figure 7B).

**Figure 8:**
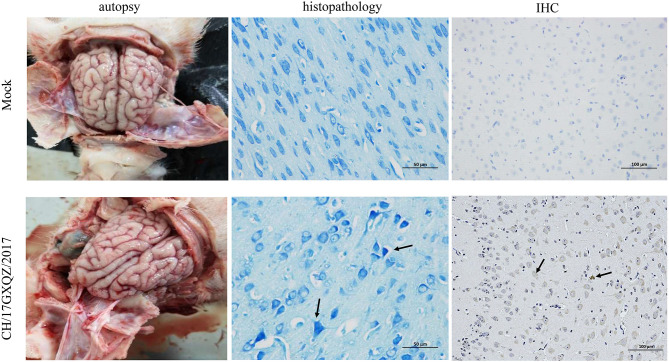
Macroscopic, histologic lesions and immunohistochemistry of the cerebrum as caused by EV-G. Macroscopic observations of the brain of one representative piglet from the mock and the inoculated groups. The microscopic lesions observed in the cerebrum of piglets at 400 × magnification with Nissl staining of a section in one representative piglet from the mock and infected groups, and the black arrows in micrograph indicate Nissl bodies vacuolization, fusion and uneven dyeing. Immunohistochemical staining of cerebrum sections from the mock and EV-G-infected groups. EV-G antigen was detected (marked with black arrows) in the brain of EV-G-inoculated pigs. The neuronal cells of EV-G-inoculated piglets were stained brown and observed at 200 × magnification.

Brain necropsy examinations of the piglets was performed and mild hyperemia was observed in the cerebrum of infected piglets. Histopathological examination showed that the degeneration and necrosis of the neurons led to an increase in nearby glial cells and vacuolization as well as fusion of the Nissl bodies. There was also uneven dyeing of the Nissl bodies and the cell nuclei were hyperchromatic (Figure 8). However, no cerebrum lesion or pathological change were found in the mock group.

According the results of IHC, the EV-G antigen appeared in the cytoplasm and cell nuclei of neuronal cells in the infected pigs. The antigen labeling was observed as brownish-yellow granules with a diffuse distribution (Figure 8), and no antigenic marker was found in the mock group.

## Discussion

In recent years, a natural recombination insertion of the PLCP gene in enterovirus was found in the United States, South Korea, Japan, Belgium and China. Most of recombinant EV-G-PLCP strains were detected from fecal samples taken from these animals indicating that EV-G-PLCP was the potential causative agent for porcine diarrhea. A previous study has shown that when the PLCP gene was inserted into the EV-G genome, it could potentially suppress the host cellular innate immune response (16). It was reported that the recombinant events which occurred in EV-G1, EV-G2, and EV-G17 genotypes are rare, but they seem to occur more frequently in EV-G1 genotype, and this might contribute significantly evolution of the virus (23). Therefore, the finding of PLCP gene insertions in EV-G can increase the potential public health threats. There was no insertion of the PLCP gene in the CH/17GXQZ/2017 strain in this study, but successive inserts of 32 A bases at a downstream region of the IRES in the 5′-UTR of CH/17GXQZ/2017 strain were found. The 5′-UTR of EV-G is important for RNA replication due to the fact that it contains secondary structural elements, although it is not clear what the effects of continuous insertion of A bases at the 5′-UTR of enterovirus would be, and this phenomenon deserves to be tested and studied further.

The VP1 gene is important for EV-G genotyping, because the main part of the VP1 protein is exposed to the surface of the capsid and it is the main component that determines the antigenicity of the virus (http://www.picornaviridae.com/) (34). A phylogenetic tree was constructed based on the EV-G VP1 amino acid sequences, and it showed that the CH/17GXQZ/2017 strain clustered into the G1 genotype with EV-G1-PLCP strains. However, the G1-PLCP recombinant strains were clustered to a separate branch based on the analysis of the complete genome sequences. Based on the whole genome sequences, the strain of CH/17GXQZ/2017 in this study had the highest sequence homology, 82.9%, with the USA strain of 13-03212. The homology value was not particularly high, but this may be a reflection of the limited amount of complete sequences uploaded in the GenBank (35).

EV-G can be propagated in BHK-21, Vero, ST, and Marc-145 cells in the presence of trypsin (4). The first EV-G strain which was isolated in the United States used Marc-145 cells until 2013 (13). The Ch-ah-f1 strain is basically considered to be the representative strain in China, but the virus has yet to be isolated (10). EV-G-PLCP recombinant viruses were originally obtained using ST cells in 2015 (16). By analyzing the growth characteristics of the CH/17GXQZ/2017 strain on Marc-145 cells, we found its growth trend to be similar to that described previously (16), with the replication cycle being relatively long and the virus titer reaching its peak at 72 hpi. However, studies on the pathogenicity of EV-G in piglets are limited. In a previous study, 2-week-old piglets inoculated orally with the EV-G1 positive fecal samples showed no clinical signs, but the virus did cause pathological changes in the cerebrum and the lungs (21). In this study, the CH/17GXQZ/2017 strain was isolated and purified, which may be beneficial in order to provide more reliable data for the animal pathogenicity experiments of EV-G.

Some enteroviruses have been shown to have the ability to infect the central nervous system and cause various effects such as paralysis and ataxia (36). In our study, piglets infected with the EV-G CH/17GXQZ/2017 strain showed some clinical neurological symptoms, such as excitement, trembling of the hind-limbs and spinning in circles. In addition, samples of the brain and spinal cord from all four infected piglets were qRT-PCR-positive for EV-G and pathological lesions were observed in the brain. EV-G antigen was also detected in the brain by immunohistochemistry. The above results all indicated that EV-G had neurotropic effects, which is consistent with a previous report (21).

EV-G is prevalent and widespread in the general pig population in the middle and eastern China (21), and the infections tended to occur early, usually within the first week after birth (37). Pigs at >20 weeks of age had a lower frequency of EV-G infection compared to pigs of <15 weeks of age, which may be due to acquired immunity as the animals age (21). When 7-day-old piglets were infected with CH/17GXQZ/2017, it caused neurological symptoms and a reduced body weight. In addition, the majority of tissues from the infected piglets carried EV-G which could be detected at 7 dpi. The CH/17GXQZ/2017 strain was isolated from piglets with diarrhea, but mild diarrhea was only observed in one of the four piglets infected with this strain. Although no piglets died after being infected with the EV-G CH/17GXQZ/2017 strain during the entire experimental infection process, it is worth noting that EV-G infection can significantly affect the weight gain of suckling piglets which is particularly concerning for pig farmers.

In conclusion, an EV-G strain, named CH/17GXQZ/2017, was isolated and purified in this study. Several techniques were used to identify the biological characteristics of infection with this strain of EV-G including CPE, IFA, replication kinetics and TEM. The phylogenetic trees based on the amino acid sequences of VP1 indicated that the CH/17GXQZ/2017 strain was clustered to the G1 genotype. The pathogenicity of the CH/17GXQZ/2017 strain was determined in 7-day-old piglets, and clinical neurological symptoms manifested as mild hyperemia and Nissl bodies vacuolization in the cerebrum. In addition, infection with the CH/17GXQZ/2017 strain significantly decelerated weight gain in suckling piglets. This study provides a reference for the biological characteristics, evolution and pathogenicity of EV-G.

## Data Availability Statement

The datasets presented in this study can be found in online repositories. The names of the repository/repositories and accession number(s) can be found in the article/supplementary material.

## Ethics Statement

The animal study was reviewed and approved by the Animal Care and Welfare Committee of Guangxi University.

## Author Contributions

KO, XM, and CY conceived and designed the experiment. XM, CY, YLu, HW, QQ, RC, ZC, and YLuo performed the experiments. KO, XM, CY, YC, ZW, and WH analyzed the data. KO and XM wrote the paper. All authors have seen and approved the final manuscript.

## Conflict of Interest

The authors declare that the research was conducted in the absence of any commercial or financial relationships that could be construed as a potential conflict of interest.
